# Ebola Virus Disease in a Humanitarian Aid Worker — New York City, October 2014

**Published:** 2015-04-03

**Authors:** Kari Yacisin, Sharon Balter, Annie Fine, Don Weiss, Joel Ackelsberg, David Prezant, Ross Wilson, David Starr, Jennifer Rakeman, Marisa Raphael, Celia Quinn, Amita Toprani, Nancy Clark, Nathan Link, Demetre Daskalakis, Aletha Maybank, Marcelle Layton, Jay K. Varma

**Affiliations:** 1 Epidemic Intelligence Service, CDC; 2 New York City Department of Health and Mental Hygiene; 3 Fire Department of New York; 4 New York City Health and Hospitals Corporation; 5 Division of State and Local Readiness, Office of Public Health Preparedness and Response, CDC; 6 Bellevue Hospital Center

In late October 2014, Ebola virus disease (Ebola) was diagnosed in a humanitarian aid worker who recently returned from West Africa to New York City (NYC). The NYC Department of Health and Mental Hygiene (DOHMH) actively monitored three close contacts of the patient and 114 health care personnel. No secondary cases of Ebola were detected. In collaboration with local and state partners, DOHMH had developed protocols to respond to such an event beginning in July 2014 (1). These protocols included safely transporting a person at the first report of symptoms to a local hospital prepared to treat a patient with Ebola, laboratory testing for Ebola, and monitoring of contacts. In response to this single case of Ebola, initial health care worker active monitoring protocols needed modification to improve clarity about what types of exposure should be monitored. The response costs were high in both human resources and money: DOHMH alone spent $4.3 million. However, preparedness activities that include planning and practice in effectively monitoring the health of workers involved in Ebola patient care can help prevent transmission of Ebola.

On October 23, 2014, NYC DOHMH was notified by Médecins Sans Frontières (MSF) that one of its physicians who had returned to NYC nine days earlier from treating Ebola patients in Guinea had an oral temperature of 100.3°F (37.9º C). The physician reported fatigue for 2 days without other symptoms (e.g., vomiting, diarrhea, cough, muscle aches, or abnormal bleeding). He reported having used prescribed personal protective equipment without a known breach and following MSF’s protocol of twice daily oral temperature checks and self-monitoring for symptoms since his return to the United States. Because of his travel and work history and symptoms consistent with Ebola, DOHMH arranged for immediate transfer by the Fire Department of New York Emergency Medical Services (FDNY-EMS) to Bellevue Hospital Center, a medical facility designated by the DOHMH and the NYC Health and Hospitals Corporation (HHC) to treat Ebola patients in NYC. DOHMH’s laboratory performed nucleic acid amplification testing on blood from the patient, and within 3 hours of specimen receipt, reported a preliminary positive result for Ebola virus on October 23; this result was confirmed at CDC on October 24.

DOHMH investigators used the date of reported onset of fatigue (October 21) to set the initial time of exposure for potential contacts. This was a decision based on knowledge about how the disease might present and an attempt to not miss any persons who might have been exposed. After interviewing the patient about his movements and contacts, DOHMH investigators identified three persons with close (i.e., direct physical) contact. Contact A was a member of the patient’s household, and contacts B and C had intermittent close contact during varying time periods after October 21. All three contacts were interviewed, evaluated for symptoms, and, under orders from DOHMH, required initial home confinement and direct active monitoring of oral temperature and symptoms. This included a daily face-to-face visit between the close contact and a DOHMH or vendor staff member, followed by a second daily face-to-face visit or telephone call. After additional evaluation and assessment, contacts B and C were released from home confinement after 10 and 12 days, respectively. Contact A was released from home confinement after 19 days. All three contacts completed direct active monitoring by DOHMH for 21 days (2); none developed signs or symptoms suggestive of Ebola. The patient was hospitalized at Bellevue Hospital Center from October 23–November 11 but released from the isolation unit on November 10 after clinical improvement and two nucleic acid amplification tests of blood for Ebola virus had negative results.

DOHMH actively monitored 114 health care personnel based on three criteria: direct patient care responsibilities, entry into the patient’s room, and handling of non-decontaminated laboratory specimens. Monitored personnel included seven FDNY-EMS workers, one from the DOHMH laboratory, and 106 at Bellevue Hospital Center. The 106 hospital workers included clinical (38), laboratory (42), environmental management (22), transport (3), and support (1) staff members. All 114 personnel reported using appropriate personal protective equipment without any known breach and were categorized as low (but not zero) risk as directed by CDC guidance of October, 2014 (2). Symptoms and twice-daily oral temperatures were reported every day by telephone to DOHMH for 21 days; no movement or work restrictions were imposed. No secondary cases of Ebola were detected among these 114 health care worker contacts. No other cases of Ebola were reported in NYC in the 42 days (two incubation periods) after the patient was first identified.

## Discussion

In response to the Ebola epidemic in West Africa, in July 2014 DOHMH began preparation for the potential arrival of imported Ebola cases with enhanced preparedness and interagency collaboration. This included enhancing surveillance to rapidly recognize and respond to a report of a patient meeting the CDC clinical and risk factor criteria for a person under investigation (3); working with hospitals to prepare to evaluate any returning traveler with symptoms consistent with Ebola; deploying the U.S. Department of Defense-developed Ebola virus assay at DOHMH’s laboratory as part of CDC’s Laboratory Response Network; and providing 24-hour per day testing, specimen packaging, and transport services (1).

Initial interagency collaboration focused on streamlining preparedness activities. The FDNY-EMS established protocols for responding to emergency telephone (911) calls involving persons with illness and a history of recent travel to an Ebola-affected country, and worked with HHC and DOHMH to perform triage on such persons. The FDNY-EMS and DOHMH also worked with John F. Kennedy International Airport Border Health Station and the Port Authority of New York and New Jersey to prepare for potentially ill travelers. After New York City designated Bellevue as a hospital to manage a patient with Ebola, HHC worked with the hospital to prepare the isolation unit and develop staffing plans for safely treating such patients. DOHMH responded to HHC drills to test and practice safe triage of persons under investigation. The Office of the Chief Medical Examiner, a division of DOHMH, developed procedures for handling the body of a person under investigation or with Ebola.

On October 3, 2014, in response to the Ebola case identified in Texas in late September 2014 (4), DOHMH activated its incident command system. The goals of incident command system activation were to 1) enhance interagency coordination and accelerate planning for health care system readiness; 2) quarantine and actively monitor close contacts of an Ebola case; 3) manage waste; and 4) conduct public outreach in NYC. DOHMH collaborated with the New York State Department of Health to assess and support the readiness of three Ebola treatment centers in NYC in addition to Bellevue. DOHMH also provided outreach to support rapid identification and isolation of persons under investigation at emergency departments and other ambulatory facilities. After the Ebola case was diagnosed in NYC on October 23, DOHMH identified contractors for disposal of medical and non-medical waste and worked with the New York State Department of Health and CDC to refine policies for identifying and monitoring people at risk for Ebola.

What is already known on this topic?

Because Ebola virus disease (Ebola) has potential to spread and has a high case-fatality rate, early identification and isolation of cases is essential. To prepare for a potential Ebola case, New York City (NYC) worked to enhance public health preparedness and interagency coordination.

What is added by this report?

The first U.S. case of Ebola diagnosed in a returning humanitarian aid worker was detected in NYC in October, 2014. Three persons who had direct contact with the patient and 114 health care workers required active monitoring. This monitoring was difficult because protocols had not been finalized prior to the identification of the case. No other persons having contact with the patient developed signs or symptoms of Ebola during the monitoring periods. No other cases of Ebola were reported in NYC in the 42 days after the patient was identified.

What are the implications for public health practice?

Interagency preparedness can help to safely and efficiently isolate and diagnose Ebola cases. Public health response to Ebola is likely to be resource intensive. Even as the West Africa Ebola epidemic subsides, it is important for public health agencies to maintain preparedness for other potential imported disease threats.

The public health response to the first case of Ebola in NYC highlighted the importance of collaboration. First, DOHMH and MSF had an established protocol for MSF to contact DOHMH when an MSF worker in NYC met the criteria for a person under investigation, and MSF required its employees to self-monitor and report an elevated body temperature or symptoms immediately. Second, beginning in August 2014, FDNY-EMS and HHC (including Bellevue) developed protocols and conducted drills on their own and with DOHMH, which permitted a person under investigation to be safely and quickly transported from home to the hospital. FDNY-EMS committed to transport of these patients only by personnel who had extensive training and experience in hazardous (chemical, biological, nuclear) materials response and had received additional training to safely and efficiently provide pre-hospital care for an Ebola patient. Third, protocols for packing, transporting, and testing specimens for Ebola virus were established among the receiving hospital, DOHMH’s laboratory, and CDC, permitting timely and efficient diagnosis. Finally, DOHMH increased public outreach efforts and, by October 31, had participated in 34 community events, contacted more than 160 West African organizations, sent community outreach teams to neighborhoods to disseminate accurate information on Ebola transmission and symptoms, and distributed 51,000 informational cards.

Despite planning and collaboration, a number of challenges remained. Creating clear and implementable criteria for health care worker monitoring based on a worker’s tasks or entry into specific zones was difficult. Persons entering the patient care room clearly required monitoring according to CDC Movement and Monitoring guidance (2). However, it was difficult to decide whether others, such as laboratory staff or waste handlers, also required monitoring. For example, “performing laboratory work” as a criterion for monitoring evolved as DOHMH and HHC discussed the exact laboratory work performed. Subsequently, workers performing laboratory work on decontaminated specimens did not require monitoring. Instituting an effective monitoring system that included timely and clear transmission of data between DOHMH and the hospital also proved difficult. Establishing protocols for workers to report oral temperatures and any symptoms to the call center took several days, and some workers had to be reminded to call DOHMH. As monitoring procedures became clearer and more efficient, worker compliance with reporting improved. Data management for worker monitoring initially required more than 12 full-time staff members of DOHMH and HHC, and managing data flow between the two agencies required close communication. Finally, there was insufficient planning on what instructions to give workers who required active monitoring if they planned to travel outside of NYC while being monitored, especially in the context of evolving local, state, federal, and international policies on movement restrictions for persons in contact with Ebola patients.

In NYC, the public health response to one Ebola case was resource intensive for a local health department, with participation of more than 500 DOHMH staff members and expenditures of more than $4,300,000 in DOHMH funds. These figures include not only the direct costs of the local public health response (e.g., contact tracing, environmental issues, and health care worker monitoring) but also the indirect costs of increasing citywide preparedness after identifying the one case (e.g., enhancing hospital preparedness, active monitoring of returning travelers, and community outreach). Ebola preparedness might include advanced planning with all designated Ebola hospitals to establish efficient monitoring programs for workers involved in caring for Ebola patients, as well as a plan for local resource allocation needed once an Ebola case has been confirmed.
